# Effect of nanogold particles addition on dimensional stability of complete denture base material: an in - vitro study

**DOI:** 10.1186/s12903-023-02850-1

**Published:** 2023-03-16

**Authors:** Esraa Yousif Taha, Moataz Mostafa Bahgat Elmahdy, Sayed Mohamed Mohamed El Masry, Mohammed Ezzat Elsayed

**Affiliations:** 1 Department of Prosthodontics, Faculty of Dentistry, Zagazig University, El-Sharkia, Egypt; 2 Removable Prosthodontic Department, Suez Canal University, Ismailia, Egypt

**Keywords:** PMMA, Gold nanoparticles (AuNps), CAD Star digital scanner, Dimensional stability, Denture base

## Abstract

**Background:**

The most widely used substance in the fabrication of dental prosthesis is poly (methyl methacrylate), or PMMA, and the development of biofilm is frequently associated with its use. To enhance the mechanical properties of heat-polymerized PMMA, this study prepared PMMA/gold nanoparticles (AuNps). The occlusal vertical dimension and tooth movement were examined in the current study. The occlusal vertical dimension was assessed using an electronic digital calliper measuring device, and tooth movement was measured using a CAD Star digital scanner.

**Results:**

Tooth movement and occlusal vertical dimension of a PMMA/gold nanoparticles (AuNps) were decreased for all groups containing AuNps. Statistical analysis was performed by means of the SPSS 16 software package.

**Conclusions:**

Incorporation of AuNps into heat- polymerized PMMA resin led to increase dimensional stability of complete denture base material.

**Supplementary Information:**

The online version contains supplementary material available at 10.1186/s12903-023-02850-1.

## Introduction

PMMA is the most popular polymer used as a denture base material due to its many benefits [1]. PMMA exhibits good aesthetic qualities, is biocompatible in the oral environment, is simple to produce, and is an inexpensive material [2, 3]. However, due to its low impact and flexural strength, dimensional instability at different thermal temperatures that can cause crack propagation, and the formation of microporosities that can result in prosthetic device fracture, PMMA lacks the mechanical properties needed to withstand the high occlusal forces [4].

The usage of polyamide-based removable partial dentures could be a viable alternative to PMMA-based removable partial dentures. Retentive features that match the colour of the gums or teeth are possible thanks to polyamide's flexibility. Clasps, minor and major connectors, and denture bases may all be put together to create partial dentures. These flexible bases may result in more somatic tissue displacement, and it is yet unclear how they affect ridge resorption. As a result, these non-rigid denture designs are contentious. A metal framework with rigid main connectors and occlusal rests integrated into the design could be a solution [5].

Complete denture management's profitability is dependent on a variety of elements, including adaptability, physiological vertical dimension, appropriate condylar position, absence of occlusal disruption, emotional concerns, and aesthetic considerations [6].

PMMA has been reinforced with a variety of substances (fillers and fibres), including metal glass fibres, fillers, wires, metal oxides, and more recently, nanofillers. For PMMA reinforcement, nanotechnology created fillers and fibres in the nanometer range. The enhancement of the mechanical characteristics of acrylic resin depends on the consistent concentration and distribution of the nanoparticles introduced, as well as the establishment of a strong link between the resin matrix and the nanoparticles. Due to the excellent biocompatibility of this metal, there are many direct uses for gold in medical devices, including wires for pacemakers and gold-plated stents that are used in the treatment of heart disease.Gold nanoparticles are currently being used in numerous technological applications and are growing in popularity, however adding Au particles to any kind of material might increase its properties, but increases its costs. [7].

Numerous studies have been conducted throughout the years to modify the polymeric structure of the acrylic resin denture base. However, the experimental effort was stopped for a number of reasons. An "effective and popular approach" of replacing missing teeth that may be the consequence of untreated caries, traumas, tumours, or congenital anomalies is dental therapy incorporating implants (hypodontia). A well-known form of treatment is the insertion of implants to replace missing teeth. It guarantees patient happiness as well as a functional, aesthetically pleasing, and long-lasting restoration. Over time, efforts have been made to reduce the length of implant treatment [8].

A revolutionary method that is increasingly being used in today's society is three-dimensional (3D) printing. It is primarily used in dentistry for dental restorations and prosthetic devices, but it may also be used to print surgical specimens such surgical guides, customised parts, and anatomical models. The component may be prepared precisely thanks to the model's precision. Regrettably, the scan and extra prints considerably raise the price of the entire process; as a result, they cannot be used in all circumstances. High accuracy, precise shape moulding, and quick print performance are just a few of the benefits and superior qualities of printable materials as compared to conventional ones. They should have great biocompatibility regardless of their intended function [9].

Recently, researchers have concentrated on using fillers with various sizes, shapes, orientations, and forms to strengthen the denture base resin. Since the development of nanotechnology, nanofillers are being utilised more frequently to improve the mechanical qualities of denture base resin. However, there is a paucity of information and comprehension regarding the efficiency of these fillers and their ideal loading in denture base resin. Therefore, the purpose of this work was to evaluate and investigate how one of the reinforcing agents, gold, affected the mechanical characteristics of the heat-cured PMMA denture base resin [7].

In the current study, gold nanoparticles (AuNps) were included. Because they possess desirable qualities like stability, non-toxicity, homogeneous particle size, and antibacterial characteristics, AuNps are an excellent option for fillers in nanocomposites. Numerous microbes, including Candida albicans, Staphylococcus aureus, Enterococcus faecalis, Escherichia coli, and/or Pseudomonas aeruginosa, have shown antimicrobial properties in particular when exposed to them. There are three common methods for combining AuNps with the polymer substance: (1) Adding nanoparticles to a polymer, (2) producing nanoparticles during polymerization, and (3) (also here) adding nanoparticles to the monomer [10, 11].

There are several studies in the literature that look at the dimensional stability of various denture base materials, but there is a dearth of information on how these AuNps affect the incorporation of denture base polymers [12, 13].

Regarding the nanoparticles, an emerging approach of choice, the Turkevich chemical reduction procedure, was used to create AuNps from gold (III) acetate (Au(CH3COO)3). Since it solves technological issues with previous approaches and produces well-defined materials with restricted size dispersion, this method has lately been advocated for scaling up the production of AuNps [14–17].

The issue with the literature is that the mechanical characteristics of AuNps-doped denture base resins and their dimensional stability have only sometimes been described combined. However, as the composite in the dental prosthesis will eventually be subjected to significant pressure during mastication, the mechanical qualities are just as crucial as the dimensional stability. The mechanical properties of PMMA may also be impacted by the addition of AuNps, as studied here. Therefore, tooth movement and the vertical dimension of occlusion were the analysed properties. The present study's most significant addition is its comparative evaluation of the impact of USP-produced AuNps on the mechanical characteristics of heat-polymerized acrylic resin [18, 19].

## Materials and methods

### Synthesis and characterization of AuNPs (gold nanoparticles) [20]

As described by Turkevich, gold nanoparticles were created using a chemical reduction process. As a precursor for gold ions (Au3 +), chloroauric acid solution (HAuCl4) has been employed. Polyvinylpyrrolidone (PVP) with a 40 K Molecular Weight was employed as a stabilising agent and sodium citrate was used as a reducing agent. As the solution's hue gradually changed to wine red, it became clear that the Au3 + ions had been converted to gold nanoparticles.

The suspension was thermally dried at 80 °C, ground into a fine powder, and then physically combined with heat-cured acrylic resin at a ratio of 0.05% weight to weight.

Eighteen identical waxed-up maxillary full dentures were made, and they were separated into two equal groups. Nine typical heat-cured acrylic maxillary dentures constituted Group (I). Nine acrylic resin maxillary dentures modified with gold nanoparticles (AuNPs) constituted Group (II).

### Denture fabrication

A silicone mould of a master edentulous maxillary cast without any irregularities on the alveolar ridge surfaces was used to create 18 identical maxillary stone casts. The casts were created using synthetic stone. On the master cast, an auto-polymerizing acrylic resin record foundation (thickness = 2 mm) was created using a procedure that has already been mentioned. In the cast's buccal sulcus, an occlusal wax rim with a height of 20 mm was made, and the height was gradually decreased to 10 mm in the second molar region. Teeth from an acrylic resin denture^1^ are put on the mould. The carved wax rim was used as a reference point to position the canine, central and lateral incisors, and other anterior teeth on the left side of the mouth. The same process was used.

Ideal tooth arrangements for complete dentures were created using two sheets of modelling wax that were modified for the cast. Using twenty sets of semi-anatomical teeth and twenty semi-anatomic teeth, the optimal tooth arrangement from the original cast was duplicated to create the tooth arrangements on all of the casts.

The occlusal surfaces of the teeth were oriented against the stone matrix until the incisal pin made contact with the incisal table on all of the maxillary waxed up dentures with their casts, in order to verify that they were all mounted at the same position and vertical dimension.

Each waxed-up denture's surface was scanned using a digital scanner, producing a file in the standard tessellation language (STL) format.

All of the dentures were processed using a standardised process in accordance with the manufacturer's specifications. The resin was polymerized using a lengthy curing cycle (74 °C for 8 h, followed by 100 °C for 1 h).

### Measurements

#### Measuring the tooth movement

GOM Inspect Professional polygonizes point clouds into high-quality 3D mesh data and offers a range of mesh processing functions. Inspection is based on a comparison of measurement data with CAD data and an analysis of false color plots, 2D sections or multiple inspection points. The free GOM Inspect software allows easy exchange and further analysis of the measurement results.

In modern industrial metrology, different measuring technologies are implemented based on application requirements. These solutions produce data for processing and evaluation, and often many different software packages are used. GOM Inspect Professional offers inspection and evaluation functions for measurement data from GOM's measuring systems, 3D scanners, laser scanners, CTs, CMMs and other sources. In order to ensure precise measurement accuracy, both GOM Inspect Professional and GOM Inspect software have been tested and certified by PTB and NIST institutions. The accuracy of the evaluation software is tested by comparing the results from the software with reference results.

Following processing, each denture was hydrated for 24 h (19) before being scanned with a CAD Star digital scanner^2^ and creating a new STL file. Surface-matching software^3^ was used to combine the STL files from the pre- and post-processing phases. By identifying 10,000 locations of overlap between the pre- and post-processing files, the global registration function enabled superimposition.

**Fig. 1:**
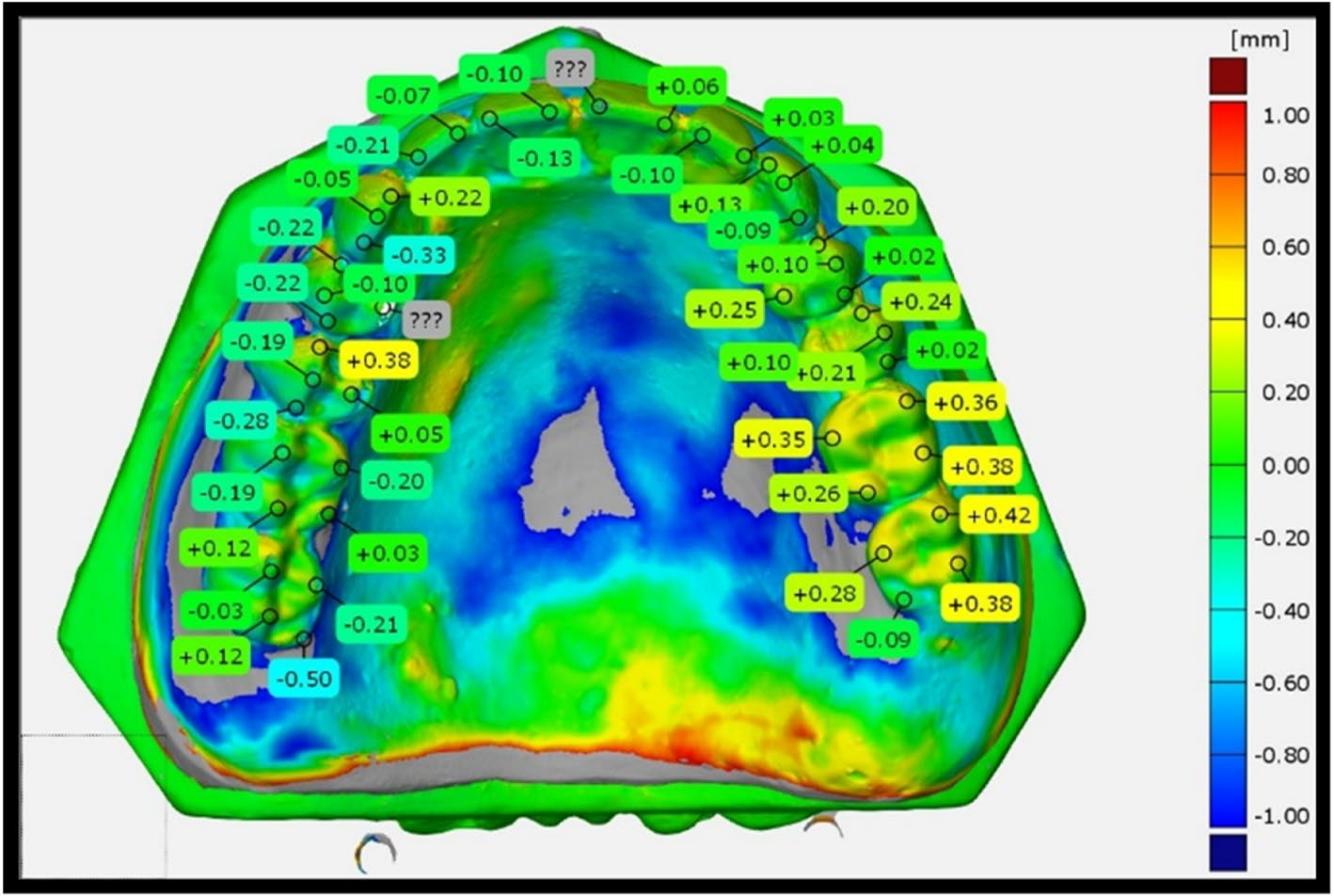
shows evaluation of tooth movement in all 46 points. * CAD Scanner, Austria. ** Gom inspect 2018, GOM, Germany

To visually demonstrate the direction and magnitude of denture tooth movement, color surface maps were made using this software's 3D comparison capability. A maximum nominal value of 0.2 mm and a maximum critical value of 1 mm were set for color spectra, respectively. In addition, all dentures had measurements taken at 46 places (Fig 1).

The surface-matching and measurements were used as the basis for evaluating tooth movement in the buccal, lingual, mesial-distal, and occlusal directions.

### Measuring the vertical dimension of occlusion

The vertical dimension of the occlusion and tooth movement were measured using an electronic digital caliper measuring device,^4^ which is capable of detecting changes as tiny as 0.01 mm.

Before processing, the distance between the articulator's upper and lower members was to be measured using a digital calliper. The articulator was put on a tabletop in a constant, specified location (initial measurement).

The occlusal plaster jig on the articulator was used to remount the dentures on the casts after processing, deflasking, and finishing.

**Fig. 2:**
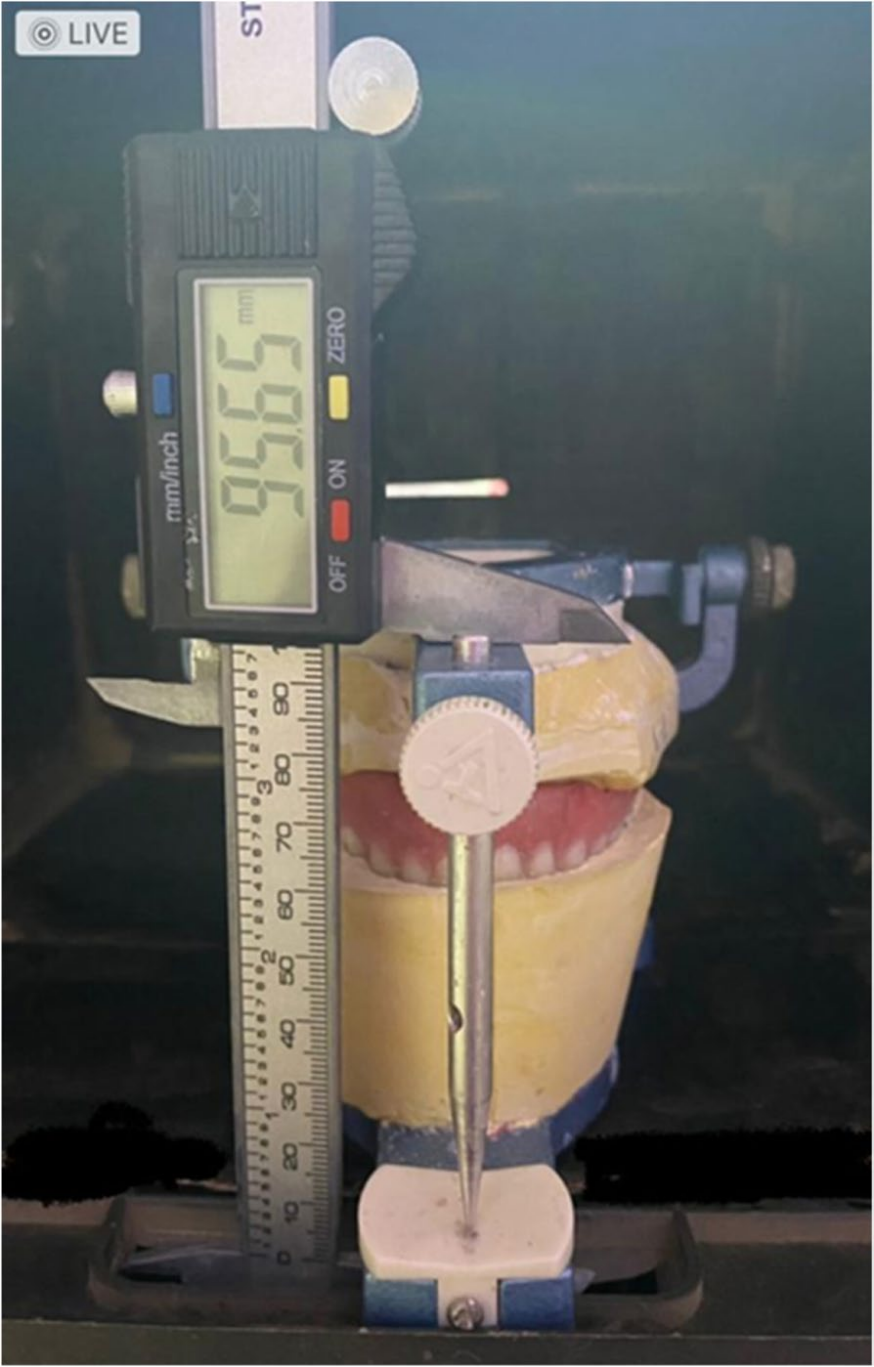
shows the digital calliper was used to measure the distance between upper and lower member of the articulator at the constant predetermined position (final measurement)

The upper and lower members of the articulator's distance were measured by the digital calliper at a fixed, predetermined position (final measurement) (Fig 2).

Two vertical measurements were taken: the first with the waxed-up denture and the second after the denture had been processed.

The variation between the final and initial measurements was related to changes in occlusal vertical dimension.

#### Data analysis

The collected data were obtained through in-vitro case–control study on randomly selected samples according to selected eligibility criteria.

Data were statistically analyzed by Microsoft Excel ® 2016,^5^Statistical Package for Social Science (SPSS)® Ver. 24.^6^and Minitab^7^ ® statistical software Ver. 16.

Data were revealed as means and standard deviations for further analysis using paired t-test to evaluate effect of processing on different dimensions of complete denture.

In addition, Student’s t test performed to evaluate significance comparison between both groups.

## Results

**Fig. 3:**
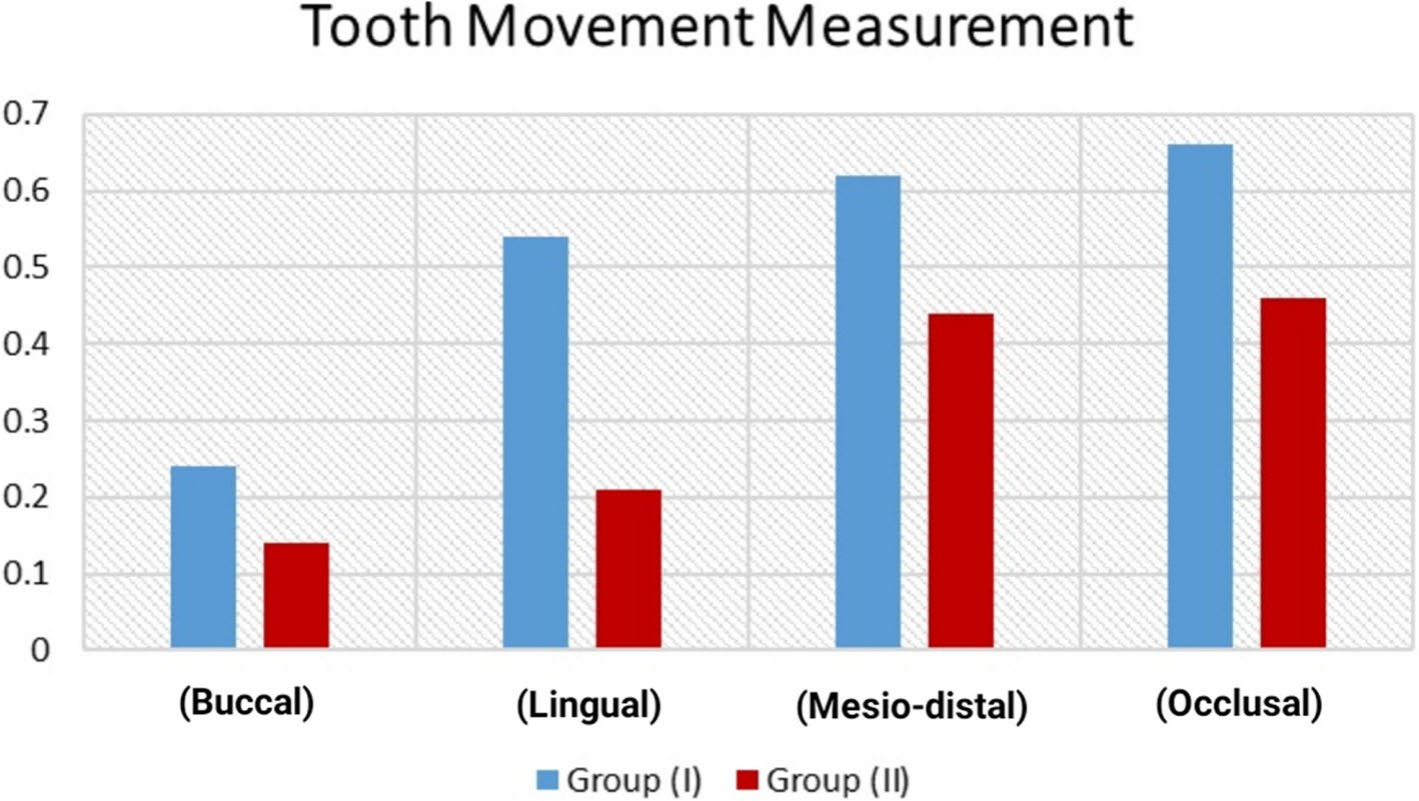
Bar chart revealing descriptive and comparative statistics of different tooth movement measurement

Using Student`s t-test for significance evaluation of independent variables, it was revealed that there was significant difference between both groups as *P*-value < 0.05, as listed in Table 1 and showed in Fig. 3.

**Table 1:** Descriptive and comparative statistics of different tooth movement measurement

	N	Group (I)		Group (II)		*P*-value
		MD	SD	MD	SD	
**(Buccal)**	9	0.24	0.001	0.14	0.07	**0.0006**^**a**^
**(Lingual)**	9	0.54	0.14	0.21	0.13	**0.0001**^**a**^
**(Mesio-distal)**	9	0.62	0.16	0.44	0.02	**0.0041**^**a**^
**(Occlusal)**	9	0.66	0.14	0.46	0.07	**0.0015**^**a**^

*N* Number, *MD* Mean Difference, *SD* Standard Deviation, *P* Probability Level

^a^significant Difference

### Vertical dimension of occlusion measurement

**Fig. 4:**
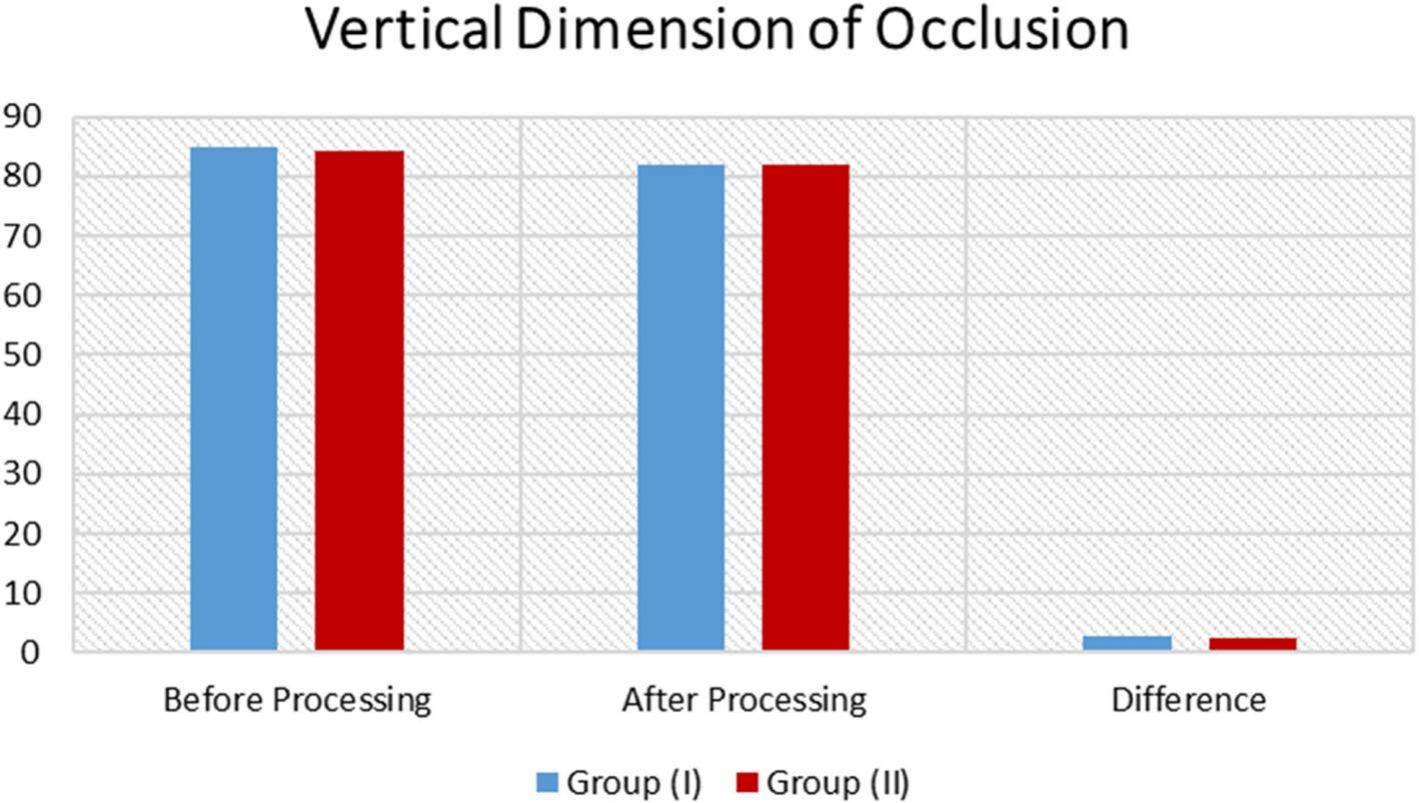
Bar Chart revealing descriptive and comparative statistics of vertical dimension of occlusion measurement

Using Student`s t-test for significance evaluation of independent variables, it was revealed that there was insignificant difference between both groups as *P*-value > 0.05, as listed in Table 2 and showed in Fig. 4.

**Table 2:** Descriptive and comparative statistics of vertical dimension of occlusion measurement

		**N**	Before Processing		After Processing		Difference (M ± SD)	***P***-value
			M	SD	M	SD		
**(VDO)**	**Group (I)**	9	84.80	1.66	82.11	0.33	2.69 ± 1.33	**0.5485 (ns)**
	**Group (II)**	9	84.30	0.10	81.89	0.43	2.41 ± 0.33	

*N* Number, *M* Mean, *SD* Standard Deviation, *P* Probability Level, *Ns* insignificant Difference

## Discussion

However adding Au particles to any kind of material might increase its properties, but increases its costs, but the goal of this study was to determine how the addition of gold nanoparticles to typical acrylic resin materials affected the materials' dimensional stability and tooth movement. For the measured qualities, the hypothesis was only partially accepted because the addition of AuNPs greatly improved the dimensional accuracy of PMMA. Because denture bases are in direct contact with the oral mucosa, biocompatible materials are necessary to prevent hypersensitivity reactions and the release of harmful compounds when used in clinical settings. The size of the AuNp particles chosen for this study was 10 and 20 nm for group 2, which is in agreement with the study that showed that gold nanoparticles with a size of 1–2 nm have very toxic effects, whereas many studies have shown that AuNPs with a size range of 14 to 100 nm have no cytotoxic effects in cells [21].

Gold nanoparticles, on the other hand, are promising antibacterial because of their large specific surface area, simplicity in functional group modification, and broad-spectrum antibacterial action. Particle size, dispersibility, and surface modification are directly correlated with their antibacterial characteristics and can be modified by varying reaction conditions. By mixing AuNPs with other metals, metal oxides, or metal hydroxides, it is possible to enhance their antibacterial capabilities. Low amounts of Au and Ag are more harmful to bacteria but less poisonous to normal cells. As a result, silver and gold composites have been extensively researched as antibacterial agents. Gold nanoparticles can be synthesised on a supporting material to enhance the antibacterial characteristics of nanomaterials. This can successfully address the issue of nanoparticle aggregation [22].

There have been conflicting findings in studies looking at how adding various nanoparticles to acrylic resin affects its mechanical qualities. However, none of the research in the literature have looked at how gold nanoparticles affect the mechanical properties of PMMA, making it impossible to compare the findings of this work to those of earlier, comparable investigations [23].

Asghari Sana et al. reported that the effect of the incorporation of nanoparticles such as Ag, TiO2, and SiO2 on the mechanical properties of acrylic resins is directly correlated with the concentration of nanoparticles, with nanoparticle strength decreasing as nanoparticle concentrations increase. This study used gold nanoparticles with a low concentration (0.05%) in accordance with their findings. A higher concentration of AuNPs (0.2%) compared to a lower concentration of AuNPs (0.05%) resulted in reduced PMMA flexural strength, according to the study. It is therefore possible to draw the conclusion that adding AuNPs to PMMA in the right concentrations may improve the mechanical properties of denture bases in clinical practice [21–25].

According to the results of a study, conventional heat-cured acrylic denture base resins showed the greatest variations in tooth position and vertical dimensions across all measurements, whereas nanoparticle-reinforced heat-cured acrylic denture base resins showed the least amount of variation in these areas. The remarkable reduction in polymerization shrinkage and the improvement in dimensional precision brought about by the addition of nanogold particles to the PMMA could be the cause of this discrepancy. The results of this investigation support those of Begum SS et. al. who found that the reinforcement of nanoparticle fillers with heat-cured denture bases increases dimensional accuracy and fits anteroposterior and mediolaterally [26].

## Conclusion

Within the limitations based on the results of this in vitro study, it could be concluded that:

1. Gold nanoparticles (AuNPs) modified heat cured acrylic resin with concentration 0.05% showed better dimensional stability than conventional heat cured acrylic resin denture base material.
2. Gold nanoparticles (AuNPs) modified heat cured acrylic resin with concentration 0.05% can decrease the tooth movement than conventional heat cured acrylic resin denture base material.

The current study only analyzes in vitro situations and an one brand of heat-polymerized PMMA; more research should be done on different varieties of the material. Following that, research on biocompatibility and antibacterial characteristics is progressing as planned, with clinical investigations as their eventual outcome.

## Supplementary Information

**Additional file 1. **

## Data Availability

The results/data/figures in this manuscript have not been published elsewhere, nor are they under consideration (from you or one of your Contributing Authors) by another publisher. The materials and data used in this study are attached to Supplemantry file 1.

## Footnotes

1 Eray, Turkey.

2 (vertex) Rapid heat polymerized conventional acrylic resin, The Netherlands.

3 (vertex) Rapid heat polymerized conventional acrylic resin, The Netherlands.

4 Digital callipers -Mitutoyo Corporation, Kanagawa, Japan.

5 Microsoft Cooperation, USA.

6 IBM Product, USA.

7 Minitab LLC, USA.
