# Supplementary material for: Effect of nanogold particles addition on dimensional stability of complete denture base material: an in - vitro study
Source: BMC Oral Health. 2023 Mar 16;23:153. doi: 10.1186/s12903-023-02850-1 (PMC10022070; doi:10.1186/s12903-023-02850-1)
Supplement: Supplementary file 1 — Additional file 1. [file 12903_2023_2850_MOESM1_ESM.docx]

**Supplementary file**

 The datasets generated and/or analysed during the current study are available in the :-

| Heat Cured acrylic Resin | Polymethylmethacrylate | (vertex Rapid heat polymerized conventional acrylic resin, The Netherlands). | 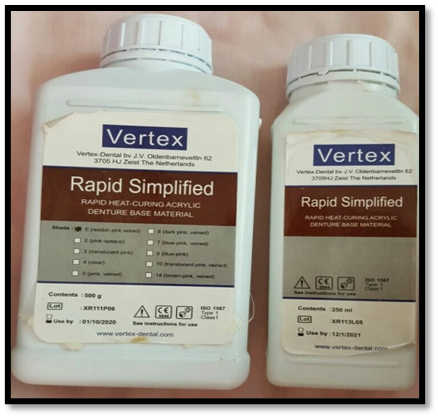 |
| --- | --- | --- | --- |
| Nano gold Heat Cured acrylic Resin | Polymethylmethacrylate with nano gold particles | (vertex) Rapid heat polymerized conventional acrylic resin, The Netherlands |  |
| nano gold wine red in color |  |  | 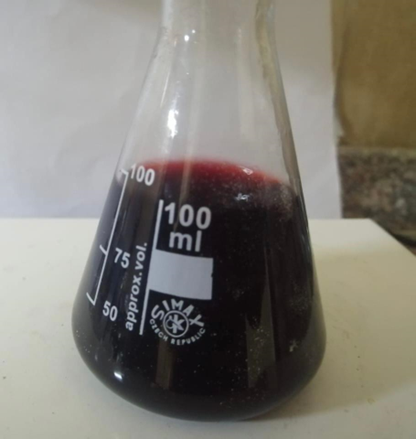 |
| readymade silicone mold. |  | RTC silicone rubber, USA. | 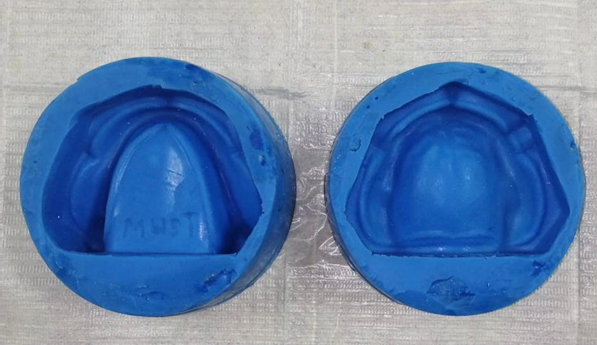 |
| Semi anatomic acrylic resin teeth |  | Eray, Turkey. | 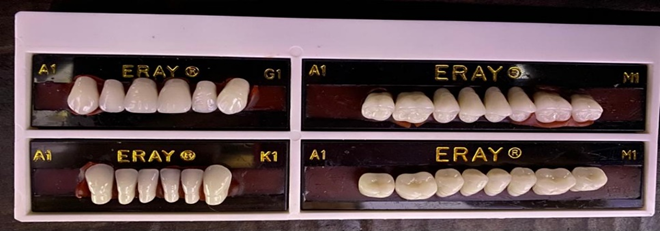 |
| special jig of dental stone matrix of the occlusal surface of maxillary master denture. |  | Fastone™ Type 3 Dental Stone | 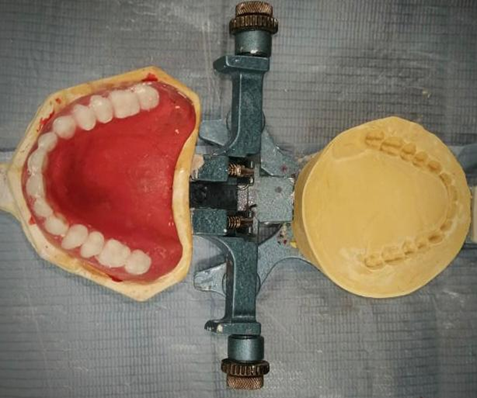 |
| electronic digital caliper measuring device. |  | ^*^Digital callipers -Mitutoyo Corporation, Kanagawa, Japan | 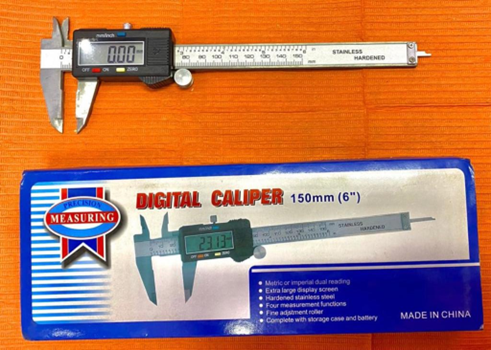 |

| the digital calliper was used to measure the distance between upper and lower member of the articulator at the constant predetermined position (final measurement) | *Digital callipers -Mitutoyo Corporation, Kanagawa, Japan. | 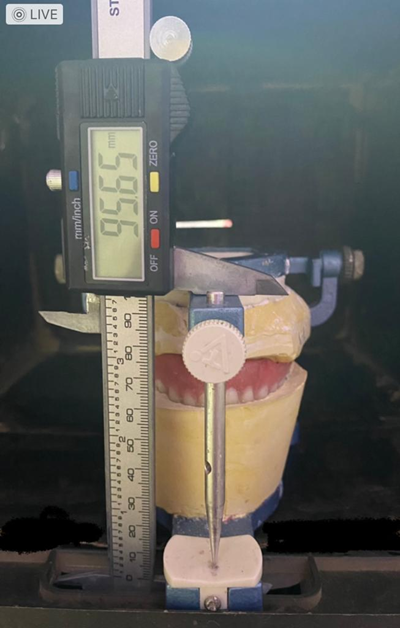 |
| --- | --- | --- |
| standard tessellation language (STL) format ﬁle of waxed up denture being scanned with a CAD Star digital scanner* (CAD Scanner, Austria.) | Surface-matching software**  ** Gom inspect 2018, GOM , Germany. | 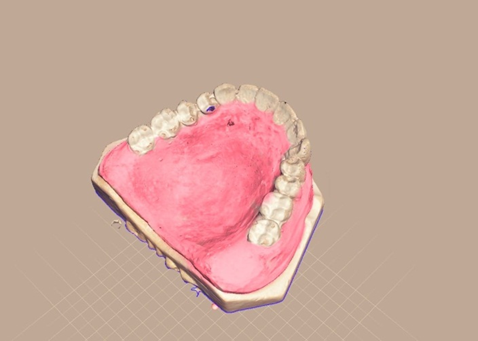 |

**Raw data**

1. **Tooth Movement Measurement:**
2. **Buccal Distance:**

Means of **Buccal** transverse distance of the samples for group (I) before and after processing were (3.19) and (2.95) mm respectively with (0.24±0.001) mm decrease in distance.

Means of **Buccal** transverse distance of the samples for group (II) before and after processing were (3.52) and (3.38) mm respectively with (0.14±0.07) mm decrease in distance.

Using Student`s t-test for significance evaluation of independent variables, it was revealed that there was significant difference between both groups as P-value < 0.05.

***Descriptive and Comparative Statistics of Buccal Transverse Distance between Both Groups:***

|  | | **N** | **Before Processing** | | **After Processing** | | **Difference**  **(M±SD)** | **P-value** |
| --- | --- | --- | --- | --- | --- | --- | --- | --- |
|  |  |  | **M** | **SD** | **M** | **SD** |  |  |
| **(buccal)** | **Group (I)** | 9 | 3.19 | 0.13 | 2.95 | 0.13 | 0.24±0.001 | **0.0006**** |
|  | **Group (II)** | 9 | 3.52 | 0.4 | 3.38 | 0.47 | 0.14±0.07 |  |

***N; Number, M; Mean, SD; Standard Deviation, P; Probability Level***

*****significant Difference***

1. **lingual Transverse Distance:**

Means of **lingual** transverse distance of the samples for group (I) before and after processing were (48.92) and (48.38) mm respectively with (0.54±0.14) mm decrease in distance.

Means of **lingual** transverse distance of the samples for group (II) before and after processing were (48.72) and (48.51) mm respectively with (0.21±0.13) mm decrease in distance.

Using Student`s t-test for significance evaluation of independent variables, it was revealed that there was significant difference between both groups as P-value < 0.05.

***Descriptive and Comparative Statistics of lingualTransverse Distance between Both Groups:***

|  | | **N** | **Before Processing** | | **After Processing** | | **Difference**  **(M±SD)** | **P-value** |
| --- | --- | --- | --- | --- | --- | --- | --- | --- |
|  |  |  | **M** | **SD** | **M** | **SD** |  |  |
| **(lingual)** | **Group (I)** | 9 | 48.92 | 0.62 | 48.38 | 0.76 | 0.54±0.14 | **0.0001**** |
|  | **Group (II)** | 9 | 48.72 | 0.44 | 48.51 | 0.57 | 0.21±0.13 |  |

***N; Number, M; Mean, SD; Standard Deviation, P; Probability Level***

*****significant Difference***

1. **Mesio-distal Distance:**

Means of **Mesio-distal** distance of the samples for group (I) before and after processing were (44.81) and (44.19) mm respectively with (0.62±0.16) mm decrease in distance.

Means of **Mesio-distal** distance of the samples for group (II) before and after processing were (44.49) and (44.05) mm respectively with (0.44±0.02) mm decrease in distance.

Using Student`s t-test for significance evaluation of independent variables, it was revealed that there was significant difference between both groups as P-value < 0.05.

***Descriptive and Comparative Statistics of* Mesio-distal *Distance between Both Groups:***

|  | | **N** | **Before Processing** | | **After Processing** | | **Difference**  **(M±SD)** | **P-value** |
| --- | --- | --- | --- | --- | --- | --- | --- | --- |
|  |  |  | **M** | **SD** | **M** | **SD** |  |  |
| **(Mesio-distal)** | **Group (I)** | 9 | 44.81 | 0.17 | 44.19 | 0.01 | 0.62±0.16 | **0.0041**** |
|  | **Group (II)** | 9 | 44.49 | 0.21 | 44.05 | 0.19 | 0.44±0.02 |  |

***N; Number, M; Mean, SD; Standard Deviation, P; Probability Level***

*****significant Difference***

1. **Occlusal Distance:**

Means of **Occlusal** distance of the samples for group (I) before and after processing were (44.96) and (44.3) mm respectively with (0.66±0.14) mm decrease in distance.

Means of **Occlusal** distance of the samples for group (II) before and after processing were (44.65) and (44.19) mm respectively with (0.55±0.62) mm decrease in distance.

Using Student`s t-test for significance evaluation of independent variables, it was revealed that there was significant difference between both groups as P-value < 0.05.

***Descriptive and Comparative Statistics of occlusal Distance between Both Groups:***

|  | | **N** | **Before Processing** | | | | **After Processing** | | | | **Difference**  **(M±SD)** | | **P-value** | |
| --- | --- | --- | --- | --- | --- | --- | --- | --- | --- | --- | --- | --- | --- | --- |
|  |  |  | **M** | | **SD** | | **M** | | **SD** | |  |  |  |  |
| **(occlusal)** | **Group (I)** | 9 | 44.96 | 0.63 | | 44.3 | | 0.49 | | 0.66±0.14 | | **0.0015**** | |  |
|  | **Group (II)** | 9 | 44.65 | 0.55 | | 44.19 | | 0.62 | | 0.46±0.07 | |  |  |  |

***N; Number, M; Mean, SD; Standard Deviation, P; Probability Level***

*****significant Difference***
